# HIV Infection Predisposes to Increased Chances of HBV Infection: Current Understanding of the Mechanisms Favoring HBV Infection at Each Clinical Stage of HIV Infection

**DOI:** 10.3389/fimmu.2022.853346

**Published:** 2022-04-01

**Authors:** Silvere D. Zaongo, Jing Ouyang, Yaling Chen, Yan-Mei Jiao, Hao Wu, Yaokai Chen

**Affiliations:** ^1^ Division of Infectious Diseases, Chongqing Public Health Medical Center, Chongqing, China; ^2^ Clinical Research Center, Chongqing Public Health Medical Center, Chongqing, China; ^3^ Fifth Medical Center of Chinese PLA General Hospital, Beijing, China; ^4^ Department of Infectious Diseases, You’an Hospital, Capital Medical University, Beijing, China

**Keywords:** HIV, HIV clinical stages, HBV, coinfection, mechanisms

## Abstract

Human immunodeficiency virus (HIV) selectively targets and destroys the infection-fighting CD4+ T-lymphocytes of the human immune system, and has a life cycle that encompasses binding to certain cells, fusion to that cell, reverse transcription of its genome, integration of its genome into the host cell DNA, replication of the HIV genome, assembly of the HIV virion, and budding and subsequent release of free HIV virions. Once a host is infected with HIV, the host’s ability to competently orchestrate effective and efficient immune responses against various microorganisms, such as viral infections, is significantly disrupted. Without modern antiretroviral therapy (ART), HIV is likely to gradually destroy the cellular immune system, and thus the initial HIV infection will inexorably evolve into acquired immunodeficiency syndrome (AIDS). Generally, HIV infection in a patient has an acute phase, a chronic phase, and an AIDS phase. During these three clinical stages, patients are found with relatively specific levels of viral RNA, develop rather distinctive immune conditions, and display unique clinical manifestations. Convergent research evidence has shown that hepatitis B virus (HBV) co-infection, a common cause of chronic liver disease, is fairly common in HIV-infected individuals. HBV invasion of the liver can be facilitated by HIV infection at each clinical stage of the infection due to a number of contributing factors, including having identical transmission routes, immunological suppression, gut microbiota dysbiosis, poor vaccination immune response to hepatitis B immunization, and drug hepatotoxicity. However, there remains a paucity of research investigation which critically describes the influence of the different HIV clinical stages and their consequences which tend to favor HBV entrenchment in the liver. Herein, we review advances in the understanding of the mechanisms favoring HBV infection at each clinical stage of HIV infection, thus paving the way toward development of potential strategies to reduce the prevalence of HBV co-infection in the HIV-infected population.

## Introduction

Human immunodeficiency virus (HIV) infection has been a major public health issue for the past four decades. Despite extensive global research and study (1–3), a cure for HIV infection has, thus far, proven elusive. Recently, our research group has proposed novel potential therapeutic options for HIV infection (4, 5) which, we believe, could inspire future clinical trials into curative therapeutic options for HIV. Our first proposition concerns the promotion of P-selectin glycoprotein ligand 1 (PSGL-1), an important receptor from innate immunity, which (i) induces the production of membrane defective virions that are unable to attach to or infect new target cells, and (ii) blocks the HIV reverse transcription process. Our second proposition involves the selective elimination of host cells capable of producing HIV virions *via* the use of a therapeutic cocktail of drugs (latency reversal agents, autophagy inhibitors, apoptosis activators, and antiretroviral drugs).

The World Health Organization (WHO) has proposed that HIV infection may be divided into four clinical stages in adults and adolescents 15 years-of-age and above (6). HIV-positive patients who are asymptomatic or have persistent generalized lymphadenopathy (lymphadenopathy of at least two sites [not including inguinal] for longer than 6 months) are categorized as being in stage 1. Clinical findings included in stage 2 (mildly symptomatic stage) are unexplained weight loss of less than 10 percent of total body weight and recurrent respiratory infections (such as sinusitis, bronchitis, otitis media, and pharyngitis), as well as a range of dermatological conditions including herpes zoster flares, angular cheilitis, recurrent oral ulcerations, papular pruritic eruptions, seborrhoeic dermatitis, and fungal nail infections. Manifestations included in clinical stage 3 (the moderately symptomatic stage) are weight loss of greater than 10 percent of total body weight, prolonged (more than 1 month) unexplained diarrhea, pulmonary tuberculosis, and severe systemic bacterial infections including pneumonia, pyelonephritis, empyema, pyomyositis, meningitis, bone and joint infections, and bacteremia. Stage 4 (the severely symptomatic stage) includes all of the AIDS-defining illnesses, e.g., HIV wasting syndrome, Pneumocystis pneumonia (PCP), recurrent severe or radiological bacterial pneumonia, extrapulmonary tuberculosis, HIV encephalopathy, CNS toxoplasmosis, chronic (more than 1 month) or orolabial herpes simplex infection, esophageal candidiasis, and Kaposi’s sarcoma. WHO HIV clinical staging utilizes standardized clinical parameters to direct medical decision making for patients with HIV/AIDS, and can be used based solely on patient clinical features, thus accommodating treatment facilities that may have limited or no access to sophisticated laboratory testing, such as those in low- and middle-income countries and regions (7). There is, also, the existence of the Fiebig staging system of HIV infection (first published in 2003, and comprising 6 stages), which describes the emergence of virological and immunological markers following infection by HIV. Several discrete clinical phases can thus be recognized for HIV infection; however, it has been generally accepted that HIV infection exhibits an acute phase, a chronic phase, and the acquired immunodeficiency syndrome (AIDS) phase (8).

In 2020, it was estimated that 36.7 million people globally were infected by HIV (9), and thus, the global HIV pandemic continues to pose a material threat to the health of mankind. The large majority of new HIV infections occur in low- and middle-income countries (10). Poverty, stigma associated with HIV disease, cultural and social barriers to appropriate testing and treatment, insufficient and inadequate health care infrastructure to support the large patient pool, poor health literacy, limited provider training, inadequate and inappropriate medical equipment, scarcity of appropriately-trained medical manpower to distribute health care throughout specific regions, and an inadequately low number of accredited medical laboratory facilities are some of the numerous factors that contribute to the almost inexorable global propagation of HIV (11).

At the same time, hepatitis B virus (HBV) is also silently spreading amongst the global population, especially in low- and middle-income countries (12). In 2019, the WHO estimated that 296 million people were living with chronic HBV (with 1.5 million new infections each year). More specifically, the WHO Western Pacific Region and the WHO African Region presents the highest chronic hepatitis B infection rates, with 116 million and 81 million people infected, respectively. Lower proportions occur in (i) the WHO Eastern Mediterranean Region (with 60 million people infected), (ii) the WHO South-East Asia Region (with 18 million people infected), (iii) the WHO European Region (with 14 million people infected), and (iv) the WHO Americas Region (with 5 million people infected) (13). Thus, HBV affects hundreds of millions of people worldwide, and is responsible for progressive liver fibrosis and hepatocellular carcinoma, amongst other chronic health sequelae (14, 15) during the chronic phase of HBV disease. Most cases of HBV infection in adults are arrested early, and are defined as an acute infection that is generally successfully limited by the patient’s own immune system. Only adults with an immunocompromised immune system tend to progress to chronic HBV (16–18). Unfortunately, most cases of HBV infection acquired in infancy or early childhood however, do become chronic (16–18). According to WHO, around one third of the world’s population has been infected by HBV at some point of their lives (16–18). Thus, HIV-HBV coinfection is relatively common (19). Estimations suggest that 10 to 28% of HIV-infected individuals are chronically infected with HBV (20–25). Indeed, the rates of HIV-HBV coinfection vary significantly between regions and risk-based groups. For instance, a study in Vietnam has shown that HIV-HBV coinfection is significantly higher among people who inject drugs (28%) or who are sex workers (15%) (23). Similarly, Xie et al. (26), have reported an estimation of 10% with respect to the existing HIV-HBV coinfection rate in China in general; however, they also state that the prevalence of such HIV-HBV coinfection in China varies between regions from 5% to 15%. In an extensive review on HIV-HBV coinfection, Singh et al. (27), suggested that West Africa and South Africa possess the highest prevalence of HIV-HBV coinfection in the world.

Several past studies have explored the impact of HIV-HBV coinfection on patients’ health, and have found that this comorbid association accelerates HBV progression (higher levels of hepatitis B viremia, higher risk of developing cirrhosis and hepatocellular carcinoma) (28), and materially amplifies the complexities related to treatment (27, 29–31). Among the mechanisms triggered by HIV infection which accelerate the progression of HBV infection, we can list (i) HIV replication in the liver, (ii) HIV-associated microbial translocation and immune activation, and (iii) immune exhaustion and tolerance. Each of these mechanisms mediated by HIV pathogenesis has significant effects on liver disease, as noted by Singh et al. (27). However, to the best of our knowledge, there remains a paucity of published research investigation in the literature which critically describes the influence and consequences of HIV clinical staging that potentially favor HBV establishment in HIV-infected individuals.

We therefore propose, herein, to review the appropriate literature to elucidate the potential mechanisms favoring HBV infection at each clinical stage of HIV infection. In the first part, we discuss the transmission routes of both HIV and HBV, and their subsequent life cycles once they have entered the human body. In the second part, we critically discuss the potential influence of each of the HIV acute, chronic, and AIDS phases that either lead to or may potentially lead to HBV infection.

## HIV And HBV: Transmission Routes And Life Cycle

**Figure 1:**
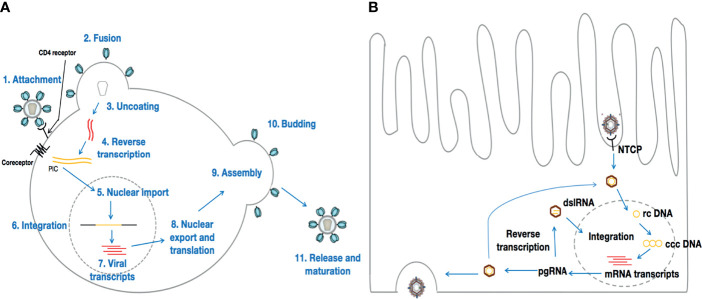
Life cycles of HIV and HBV. **(A)** represents HIV life cycle once in contact with CD4+ T-cells. Although HIV preferentially infects CD4+ T-cells, HIV tropism is not limited to CD4+ T-cells only. Conversely, HBV [the life cycle of which is depicted in **(B)**] infects hepatocytes exclusively.

It is well-established that HIV and HBV share the same transmission routes. Indeed, both viruses are known to be transmitted from person to person through sexual intercourse, *via* contaminated needles used for intravenous drug delivery, from mother to child, and by the therapeutic use of HIV or HBV-infected blood or blood products (32). Thus, individuals who have casual sex in the absence of a condom and those who inject recreational drugs are at a particularly high risk for acquiring not only HIV infection, but also HBV infection (29). Once a person is infected by either HIV or HBV, these viruses exhibit two distinct life cycles within the infected persons body (**Figure 1**).

HIV targets immune cells, preferentially CD4+ T-lymphocytes. Then, a viral envelope glycoprotein molecule (gp120) binds to a host cell receptor or co-receptor, such as CCR5 or CXCR4, responsible for HIV entry into lymphocytes and macrophages. The binding of gp120 to these receptors results in a cascade of molecular conformational changes and the exposure of gp41, bringing the HIV virion in much closer proximity to the target cell. Subsequent fusion of the viral envelope with the host cell membrane is essential for the entry of the inner matrix core of the virus into the intracytoplasmic realm of the host cell (33). Within the viral inner core are two strands of viral RNA held together by two small proteins (P6 and P7), and three of the enzymes essential for viral replication, viz., integrase, protease, and reverse transcriptase. Accessory proteins such as Nef, Vpr, and Vif are also found in the core matrix of the virus. Although these accessory proteins are not essential for viral replication, they play crucial roles in counteracting defensive mechanisms activated by the host cell (34, 35). Once within the host cell cytoplasm, the core matrix of the virion disintegrates, releasing the viral capsid as well as the genome of the virus. The viral RNA, together with the essential viral enzymes, is thus exposed to the host cell cytoplasm. The viral RNA then undergoes reverse transcription into viral DNA through a process mediated by the viral reverse transcriptase. Earlier investigations have revealed that the viral DNA generated by the reverse transcription process within the host cytoplasm is part of a broad nucleoprotein complex known as the pre-integration complex (PIC) (36), which also comprises Vpr and the integrase enzyme. Subsequent migration and entry of the PIC into the nucleus is followed by the process termed integration, which is mediated by the integrase enzyme. The preceding view, that conversion of the HIV RNA genome into DNA occurs in the cytoplasm before nuclear entry has, however, been challenged recently. Indeed, Dharan et al., have provided evidence to support the hypothesis that reverse transcription and uncoating can occur in the nucleus of non-dividing cells, such as macrophages or cells treated with the tetracyclic antibiotic, aphidicolin (37). For integration to occur, the integrase within the PIC acts by slicing through the DNA of the host cell, and thus allowing viral DNA to be inserted at a variety of sites on the host DNA, i.e., integrase catalyzes the insertion of viral dsDNA into the host chromosome (38, 39). For HIV, and most viruses that integrate into the host genome (e.g., murine leukemia virus, Herpes simplex virus-1, Ebola virus), observations from past studies (40, 41) reveal evidence of the DNA splicing and joining steps. It is critical to keep in mind that, usually, two nucleotides are removed from 3’ end of the viral DNA. Then, these 3’ ends attack a pair of phosphodiester bonds on opposite strands of the target DNA, across the major groove, leading to a bonding of the covalent 3’ ends of the viral DNA to the target DNA. Finally, the single-strand gaps and the two-nucleotide overhang at the viral DNA’s 5’ ends are repaired by cellular enzymes, in order for integration to be complete. For HIV, the sites are five base pairs apart instead of two, resulting in a five base-pair duplication (42). Once HIV DNA is integrated into the host cell genome in this manner, the host cell is considered to be infected for life. Thus, the integrated viral DNA, referred to as provirus, can be used to generate genomic RNA, which can serve as messenger RNA (mRNA) for the synthesis of viral proteins in the host cytoplasm (**Figure 1A**).

HBV targets and replicates solely in the parenchymal cells of the liver (the hepatocytes) (43–46) (**Figure 1B**). Moreover, it has been established that HBV infects only humans, chimpanzees, and to a lesser extent, tree shrews (Tupaia belangeri) (47, 48). Once in contact with the liver, the circulating virion initially attaches to heparan sulfate proteoglycans (HSPGs) (49, 50). Then, the interaction of a specific domain of the HBV L envelope protein with the sodium taurocholate co-transporting polypeptide [NTCP, a hepatocyte-specific transporter of bile acids that is predominantly localized in the basolateral membrane that faces the sinusoidal lumen (51)] on the surface of the hepatocytes contributes to viral entry into the hepatocyte (52). Following entry and uncoating, the nucleocapsid carrying the HBV genome is transported into the nucleus, where it is released as relaxed circular (rc) DNA. There, the rcDNA is converted into an episomal covalently closed circular (ccc) DNA minichromosome by host enzymes (46, 53). Reports suggest that cccDNA is very stable, persisting indefinitely, and is one of the main barriers to cure for hepatitis B disease (46), as it is the template for all HBV RNA transcripts (27, 54) that leave the nucleus unspliced, and produces the viral structural and non-structural proteins (53). Thus, HBV can initiate viral replication with an estimated doubling time of 2-4 days (55, 56). Interestingly, HBV polymerase can encode the pre-genomic RNA (pgRNA) and the reverse transcription of pgRNA can also lead to the formation of double stranded linear HBV DNA (dslDNA). Once in the nucleus, the dslDNA, in a similar manner to HIV, can integrate into the host genome (27). In contrast to HIV, the integrated dslDNA cannot enable viral replication, but it does allow the expression of certain gene products, like the envelope proteins (Env), which are dissimilar to the envelope proteins generated from cccDNA, which coat filamentous and spherical subviral particles (SVPs) (54). In general, acute manifestation of HBV infection occurs within 6 months after a person is exposed to HBV (57). From an acute infection, it can subsequently progress into a chronic infection. Indeed, although most people with healthy immune systems are able to clear the virus at the acute stage, immature immune systems and/or impaired immunity can lead to the establishment of chronic HBV infection in infants and/or adults (58, 59). Once the disease becomes chronic, it becomes a lifelong infection which, in the absence of effective treatment, can cause liver cancer or significant liver damage and scarring, leading to eventual liver failure.

*In vitro* and *in vivo* reports suggest that HIV can also infect hepatic stellate cells, sinusoidal endothelial cells, Kupffer cells, and the resident macrophages of the liver [as reported by Chamroonkul and Bansal (60), Housset et al. (61) and Cao et al. (62)]. HIV RNA sequences from the livers of untreated HIV-positive individuals show distinct compartmentalized sequences when compared to RNA sequences from other tissue sites (63). Further studies have demonstrated that HIV can persist in the liver even in patients on antiretroviral therapy (ART), primarily in Kupffer cells (64–66). In this review, therefore, we explore and discuss the influence of HIV infection on the establishment of HBV infection, especially being cognizant of the fact that HIV is known to provoke the fundamentally profound immune system impairment necessary for the onset of chronic HBV. Normally, most people with healthy immune systems are able to clear HBV during the acute phase. Utilizing the combined actions of HBV-specific CD4+ T-cells [essential for the induction and the maintenance of both CD8+ T-cells and antibody responses (67, 68)] and HBV-specific CD8+ T-cells [which kill infected hepatocytes and induce local production of proinflammatory cytokines (69–71)], a healthy person can easily overcome acute HBV infection, and thus avoid the chronic and life-threatening phase of the infection. Subsequently, our discussions will consider HIV as the primary infection, and we reflect further on the immunological consequences of HIV infection that favor HBV infection.

The various mechanisms through which liver injury may occur in patients with HIV infection are numerous; a general breakdown of these mechanisms is presented in **Table 1**. A reasonable understanding of these mechanisms is of significant importance to the comprehension of HIV/HBV pathological processes, and any liver injury may further represent an ‘open door’ for HBV to enter hepatocytes and subsequently establish infection. This preceding assertion is speculative at this stage, and further investigation is required to establish precisely how liver injury induced by HIV infection could facilitate HBV invasion of hepatocytes.

**Table 1:** Summary of reported mechanisms responsible for liver injury in patients with HIV.

Mechanism	Contribution	Details	References
Oxidative stress	Moderate	This is a process whereby free reactive oxygen species (ROS) provoke increased activation of Kupffer cells in the liver. In turn, these activated immune cells promote stellate cell activation *via* nuclear factor kappa-beta (NF-kB) and activator protein 1, leading to increased production of proinflammatory and profibrotic cytokines, resulting in liver damage, fibrosis, and cirrhosis. Nucleoside reverse transcriptase inhibitors (NRTIs) such as didanosine can cause oxidative stress and mitochondrial toxicity.	(21)
Mitochondrial injury	Moderate	As the primary source of energy in the hepatocyte, any process that impairs mitochondrial function may lead to hepatic injury. During HIV, mitochondrial injury can occur through increased stress on the endoplasmic reticulum (ER), initiated by activation of the IRE1/TRAF 2 (Inositol Requiring 1/TNF receptor-associated factor 2) pathway. NRTIs and protease inhibitors (PIs) can directly cause mitochondrial toxicity.	(21, 72, 73)
Immune-mediated injury	Moderate	HIV can interact with hepatic stellate cells (HSCs) *via* gp120, producing inappropriate activation and increased HSC production of collagen and monocyte chemoattractant protein (MCP-1) (a macrophage chemoattractant). HIV decreases the number of Kupffer cells in the liver, decreasing the ability of the liver to clear products of microbial translocation. HIV provokes alterations in cytokine profiles resulting from imbalance between CD4+ and CD8+ T-cells	(21, 74, 75)
Cytotoxicity	Mild	HIV triggers apoptosis *via* the HIV gp120 protein-receptor signaling pathway.	(76)
Systematic inflammation	Significant	The systematic inflammation resulting from HIV infection may induce fibrosis *via* a number of mechanisms, including oxidative stress and mitochondrial dysfunction as a result of ER stress. CD4/CD8 imbalances seen in HIV can lead to underexpression of IFN-gamma (an antifibrotic cytokine), thus favoring induction of apoptosis of activated HSCs, and hepatic progression into a profibrotic state.	(21, 77, 78)
Gut microbial translocation	Significant	This leads to hepatic injury primarily *via* increased hepatic levels of bacterial lipopolysaccharides (LPS), causing hepatic inflammation. More specifically, hepatic inflammation may result from (i) recruitment and activation of inflammatory cells (Kupffer cells and HSCs), (ii) systemic immune responses promoting hepatocyte cell death, or (iii) production of proinflammatory cytokines and acute phase reactants such as transforming growth factor beta 1 (TGFB1), IL-6, and IL-10	(79–81)
Nodular regenerative hyperplasia	Significant	This is a rare condition in which diffuse transformation of the liver parenchyma into micronodules without intervening fibrosis leads to non-cirrhotic portal hypertension in patients with HIV. Pathophysiologically, it is thought that gut bacterial translocation may be responsible for vascular endothelial disruption, vascular and peri-vascular fibrosis and stenosis, and portal hypertension. The epithelial damage observed in the liver isare thought to either be immune-mediated or possibly related to direct viral damage by HIV.	(82–84)

## Acute And Early HIV Infection

### Innate Immune Defense Subversion

**Figure 2:**
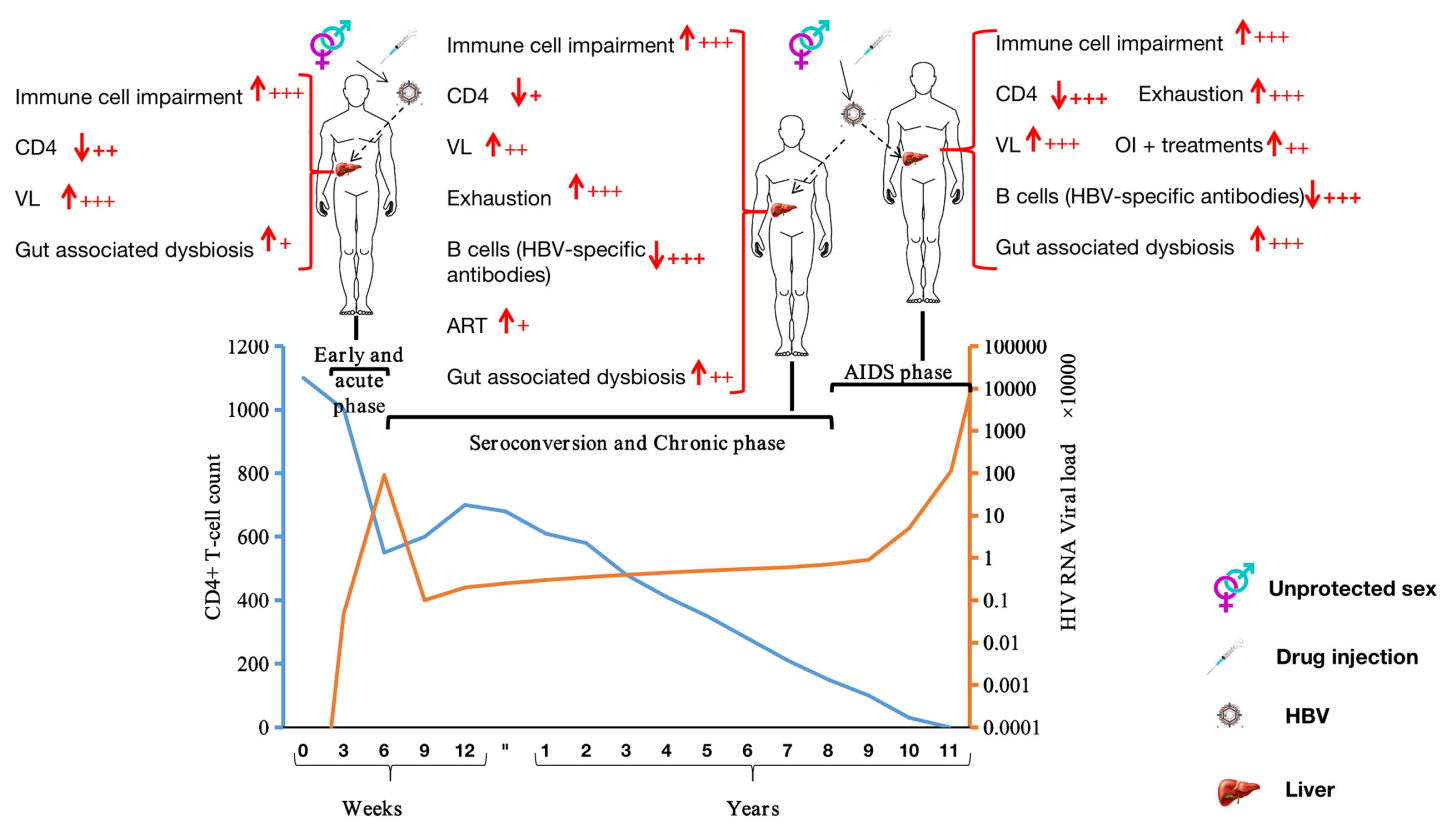
Stages of HIV infection and factors potentially favoring HBV infection at each clinical stage of HIV infection. ↓: depletion; ↑: augmentation; VL, viral RNA load; OI, opportunistic infection; +: Mild; ++: Moderate; +++: Severe.

Acute HIV infection (AHI) is the first stage of HIV infection, occurring soon after viral acquisition and before seroconversion. AHI typically lasts 3–4 weeks (**Figure 2**), and is characterized by the presence of HIV RNA and p24 antigen (Ag) (85) in the circulation. During this short period, HIV concentrations in blood and other body fluids (vaginal secretions and semen) are exceptionally high, increasing the likelihood of HIV transmission (85–92). To reach the high levels of HIV observed, HIV-1 subverts dendritic cell and macrophage activities (preferentially CD4+ T-cells) to increase its replication at mucosal locations (93, 94). This strategy also favors HBV, which does not need to use any specific mechanisms to avoid such immune cells (dendritic cells, macrophages, and T-cells) in an HIV-positive individual. Moreover, HIV adopts a variety of strategies to avoid type 1 interferon (IFN-1) control [repression of HIV restriction factors (95–101) and/or blocking of IFN-1 expression by infected cells (102–105)] *via* the infected-cells (dendritic cells, macrophages, and T-cells). Indeed, IFN-1 is an innate antiviral defense cytokine, and is known as a pleiotropic cytokine that acts by up-regulating transcription of hundreds of IFN-stimulated genes, including HIV restriction factors (106). To illustrate this point, Gondim et al. (107), for instance, have investigated how IFN-1 can control HIV infection and they have shown that IFN-1 (including IFNα2 and IFNβ) administration can reduce viral replication in CD4+ T-cells and macrophages. Furthermore, three major points depicting the interplay between HIV infection and IFN-I are of particular interest, viz., (i) the sensitivity of HIV-1 isolates to IFN-I inhibition consistently changes over time, (ii) HIV-1 isolates obtained during ART therapy were relatively IFN-I sensitive, and (iii) the viruses that rebounded after treatment interruption displayed the highest degree of IFNα2 and IFNβ resistance. Thus, IFN-1 plays an essential role in inflammation, immunoregulation, tumor cell recognition, and T-cell responses. In the absence of effective expression of IFN-1, or in the absence of response resulting from its expression, the immune system becomes vulnerable to viral infections, such as infections by HBV. In addition to IFN-1, HIV avoids INF-gamma (IFN-γ, a type II interferon) control by (i) destroying CD4+ T-cells [also responsible for IFN-γ secretion (67)] or (ii) repressing, for instance, PSGL-1 (an HIV restriction factor) activities, which has been extensively reviewed by our research group (4). Besides, in order to reduce the protective benefits of innate responses, HIV-1 resists well-demonstrated control by natural killer cell (108–113) [stimulated by innate cytokines including IFN-1, IL-15, IL-18 and receptor-ligand interactions (93)], and may disrupt innate regulation of adaptive responses, as suggested by Borrow (93). By utilizing these mechanisms, HIV infection contributes to HBV evasion of immune cells (particularly effector cells) to establish chronic HBV disease, as described by Lannacone and Guidotti (54).

### High HIV Viral Load and CD4+ T-Cell Depletion

During AHI, the elevated concentration of viral particles in the systemic circulation facilitates infection of the liver by HIV, which in turn promotes multiple pathways that all converge on activated hepatic stellate cells (HSCs), the primary source of collagen synthesis in the injured liver, which encourages hepatic inflammation and fibrosis (60). For instance, it is known that HIV and its envelope gp120 (i) promote direct pro-fibrogenic effects on HSCs, (ii) promote the production of pro-inflammatory cytokines (such as MCP1, IL-8), and (iii) induce apoptosis in hepatocytes (75, 114). Indeed, HIV glycoproteins induce hepatocyte apoptosis *via* the expression of the TNF-related apoptosis inducing ligand (TRAIL), by stimulation of hepatocytes (115, 116). Furthermore, rapid fibrosis, in addition to causing elevated plasma HIV levels, correlates with reduced CD4+ T-cell counts.

During AHI, there is extensive CD4+ T-cell destruction (HIV-induced CD4+ T-cell depletion) (93). On the one hand, this HIV-induced depletion of CD4+ T-cells relative to CD8+ T-cell recruitment alters the hepatic cytokine profile, establishing a fibrogenic environment. Consequently, an injured liver becomes an ideal target for HBV to establish an acute phase, which progressively metamorphoses into a chronic infection due to the persistence of the systemic inflammation caused by HIV infection. On the other hand, it is recognized that host CD4+ T-cells are essential for the recognition of viral antigens presented by Kupffer cells and the regulation of the activities of (i) CD8+ cytotoxic T-cells, (ii) antibody-producing B-cells, and (iii) cytokine-secreting cells (19, 117–119). When the HIV acute phase leads to drastic depletion of CD4+ T-cells, the immune system is unable to adequately respond to HBV invasion, as HBV antigens presented by Kupffer cells cannot thus be recognized. Moreover, CD8+ T-cells, B-cells, and cytokine-secreting cellular functions are overwhelmed by HIV subvertive activities, which thus facilitates HBV infection establishment.

## HIV Seroconversion And The Chronic Phase

During the seroconversion phase, which occurs after the acute phase (**Figure 2**), the body starts producing detectable levels of HIV-specific antibodies. A seropositive individual may have flu-like symptoms, such as fever and body aches during this phase. The duration for HIV disease progression with clinical symptoms varies widely across individuals, although it usually progresses slowly (120). Most HIV-positive individuals are diagnosed during or after the seroconversion phase [as HIV diagnostic tests generally target HIV-specific antibodies (121)]. During this period, the earlier detection and earlier initiation of appropriate treatment leads to a reduced risk of onward transmission. Due to HIV-specific antibody production, HIV-infection is stabilized at this stage of the infection, meaning that the plasma viral RNA load, despite being high, remains stable, CD4+ T-cells counts increase slightly, and the immune system activation remains persistent. HIV causes several structural, functional, and immunological impairments, resulting from a persisting underlying chronic inflammatory state (122–124). HBV establishment is likely to be favored by HIV infection during the seroconversion and the chronic phases as HIV infection sustains the immunological impairments present during the acute phase, in conjunction with other mechanisms, as described in the following paragraphs.

### HIV-Associated Gut Dysbiosis

It has been reported that the gastrointestinal tract (GI) represents the primary site of HIV replication and reservoir persistence (125). Once HIV infection is established, a rapid loss of GI mucosal integrity is noted. Indeed, HIV disrupts the lymphatic system of the gastrointestinal tract, causing a large loss of CD4+ T-lymphocytes in the gut-associated lymphoid tissue (GALT), which disrupts the tight junctions of the intestinal epithelium. Subsequently, this detrimentally alters the integrity of the intestinal mucosal barrier, leading to intestinal microbiomic disorders (126, 127), which manifest as a decrease in gut microbiotic organism diversity, the augmentation of specific species of potentially pathogenic gut microbiomic microorganisms (128), and the promotion of an increased permeability (or “leakiness”) of the intestinal tract. Consequently, harmful bacteria and their products, such as lipopolysaccharide (LPS), *via* their passage through the portal vein into the liver, may activate the liver’s innate immune system by recognition of Toll-like receptors (TLRs, especially TLR2 and TLR4) (129). Some investigators believe that the levels of translocated microbial products, such as LPS, in the portal vein and/or in the liver (which are both difficult to measure) may be more important than these microbial products being present in the systemic circulation (27, 54). This innate immune response, generated by pathogen-associated molecular patterns (PAMPs) produced by intestinal microbes, may be responsible for hepatocyte damage (130). To further illustrate this point, in a study by Evans et al. (131), using SIV-infected macaques, it was demonstrated that increased microbial load in the liver may also trigger chemokine production and an increased infiltration of CXCR6+ activated NK cells, known for their role in the development of liver fibrosis. An HIV-positive individual displaying an HIV-associated gut dysbiosis profile can, thus, readily develop HBV infection, as HIV-associated microbial translocation favors hepatocyte injury. Our group has recently published an extensive review discussing mechanisms whereby gastrointestinal microbiome dysbiosis and a “leaky” gut in PLWH increases susceptibility to HBV infection (132).

### Immune Cell Exhaustion

CD8+ T-cells (levels of which remain elevated in the bloodstream during HIV infection), HIV-associated dysbiosis *via* microbial translocation (128, 133), and TRAIL [a proapoptotic ligand with an immune effector function promoting the eradication of infected or malignant cells (134)], are some of the identified factors responsible for CD4+ T-cell depletion. CD4+ T-cell depletion is also responsible for liver injury, which facilitates liver invasion by HBV (as described in the preceding section). Since CD4+ T-cells are important for the recruitment of HBV-specific CD8+ T-cells, a sustained CD4+ T-cell depletion restricts the ability of the immune system to adequately and appropriately respond to HBV invasion. Indeed, in such a context, it is difficult for the immune system to locate and recruit HBV-specific CD4+ T-cells (55), which represents an essential facilitator for the induction and maintenance of both CD8+ T-cells and for B-cell antibody responses (68). Researchers have also noted exhaustion signatures in HIV-infected innate immune cells, rendering them less potent at responding not only to HIV, but also to HBV, which is inherently highly efficient at avoiding recognition by the innate immune system, as reported in several studies (135–138). For example, Wang et al., have identified exhausted CD4+ T-cells and CD8+ T-cells, and then, a closer look at the exhausted CD8+ T-cells has indicated that they present less effector function phenotypes than normal CD8+ T-cells (139). Indeed, Wang et al., have identified key upregulated genes [killer cell lectin-like receptor subfamily G member 1 (KLRG1), cluster differentiation (CD160), and T-cell immunoreceptor with Ig and ITIM domains (TIGIT)] that are associated with T-cell exhaustion. Additionally, Nguyen et al. (140), have demonstrated that HIV-specific CD8+ T-cells from the lymph nodes of HIV chronic progressors preferentially express exhaustion signatures [TIGIT, lymphocyte-activation gene 3 (LAG3), CD244 (recognized as inhibitory receptors), KLRG1, and the transcription factor EOMES (Eomesodermin, also known as T-box brain protein 2, Tbr2)] (141–143). Thus, subsequent to HIV infection, remaining CD4+ T-cells and circulating CD8+ T-cells, should they be exhausted, are potentially less potent at assuming essential protective functions compared to normal CD4+ and CD8+ T-cells. A blockade of PD1 (144), CTL-4 (144), KLRG1 (139), for example, may be potentially helpful in effectively restoring the protective functions of exhausted immune cells, which in turn could promptly respond to HBV invasion.

### Antiretroviral Treatment (ART)

Since most HIV-positive individuals are diagnosed during or after the seroconversion phase, most HIV-infected patients often initiate ART during or after this phase of the infection. ART efficiently suppresses HIV-1 replication by targeting key mechanisms in its life cycle (145), which in turn (i) reduces HIV viral RNA load to below detectable levels (146, 147), (ii) increases the circulating number of CD4+ T-cells (148, 149), (iii) reduces the incidence of AIDS-related diseases and/or deaths (148, 150), and (iv) effectively prevents the transmission of HIV to the uninfected population (151). Compared to untreated patients, ART reduces rates of hepatic fibrosis in treated patients by effectively increasing CD4+ T-cell numbers. However, active monitoring for ART-induced liver injury should be considered as it has been reported that some ART therapeutic drugs may be toxic to the liver (152, 153). Moreover, it has also been reported that liver-related death is the leading cause of non-AIDS death in patients whose HIV infection is well-controlled by ART (154). Thus, in ART-treated patients, the risk of liver injury does not originate solely from the prevalent HIV RNA viral load or from CD4+ T-cell depletion, but may also result from toxicity associated with ART drugs. This may also represent a potential additional factor facilitating HBV establishment.

### HBV Vaccinated Individuals

In people who have received the HBV immunization, the risk for developing HBV remains, as memory B-cells and long-lived plasma cells, recognized as pivotal for maintenance of serological memory to vaccines and infections, have been shown to be reduced in number during HIV-1 infection (155, 156). Interestingly, their numerical decline correlates with reduction of antibody (Ab) titers against childhood vaccinations (157, 158). It is, therefore, reasonable to speculate on the reduction of HBV-specific antibody titers subsequent to memory B-cell reduction, even if it has been demonstrated that ART initiation shortly after HIV infection may restore memory cell numbers to physiological levels in HIV-1-infected children and adults (159, 160). Moreover, exhausted memory B-cells [activated memory B (AM) and tissue-like memory (TLM) B cells)] are expanded in the circulation during HIV-1 infection (161, 162). From the investigations of Wang et al. (139), and Nguyen et al. (140), it is now known that HIV-related exhausted T-cells become less potent at accomplishing their full repertoire of immune functions. Although some clarification remains to be elucidated in this specific subject area, we may relatively confidently assume that due to HIV infection, exhausted B-cells do become dysfunctional as well, and are thus, not as immunologically competent as normal B-cells at producing specific antibodies. Chiodi and Scarlatti (163) have proposed that the B-cell dysfunctional profile (inhibition of both B-cell proliferation and antibody production) due to cellular exhaustion caused by HIV infection, could be explained by a specific pathway engaged *via* the expression of inhibitory receptors on the surface of TLM B-cells during HIV-1 infection, which includes the inhibitory receptor Fc receptor-like-4 [FCRL4, which is increased in B-cells during HIV-1 (164) infection, and acts by dampening B-cell receptor (BCR) signaling]. Furthermore, presence of IL-6, known to be increased in B-cells during HIV-1 infection, may lead to aberrant B-cell differentiation (164, 165). In such contexts, the liver is vulnerable to HBV invasion, since the expected specific antibody generation resulting from administration of the HBV vaccine would have been somewhat neutralized *via* B-cell destruction and secondary B-cell functional impairment directly attributable to HIV infection.

## Acquired Immunodeficiency Syndrome (Aids) Phase

The global success of ART in treating HIV infection and AIDS has led some to some doubt whether a curative solution to AIDS is necessary. Only patients not on ART or those who are infected with HIV strains resistant to ART can progress to the AIDS phase of HIV infection (166). In general, in untreated people or inadequately treated people, it takes several years to gradually progress from primary HIV infection to the AIDS phase, which is characterized by the onset of symptoms and signs of severe HIV illnesses and profound immunosuppression. The immunological and other issues encountered during the acute and the chronic phases of HIV infection are significantly exacerbated in the AIDS phase. A patient at this stage of the infection may have a substantially high viral load, which may, in addition to a very low CD4+ T-cell count (**Figure 2**), lead to further liver injury, thus favoring HBV infection. The overtly symptomatic stage of HIV illness denotes the late stage of HIV disease (AIDS) in which patients (i) have a CD4+ T-cell count of less than 200 cells/mm^3^ and (ii) are vulnerable to additional opportunistic infections (OIs) (167) (such as infections by *Mycobacterium avium* complex, *Mycobacterium tuberculosis*, *Pneumocystis jirovecii*, *Cytomegalovirus*, *Toxoplasma gondii*, and *Candida* species) or the occurrence of aggressive forms of Kaposi’s sarcoma or B-cell lymphoma (32). Unfortunately, numerous OIs are known to be associated with liver injury, which is a vital facilitator for HBV invasion of the liver (168–173). The liver is frequently affected by opportunistic infections, most commonly in infections by mycobacteria and *Cytomegalovirus* (174). Compared with non-TB HIV-infected patients, TB-HIV co-infected patients present with more significantly aberrant liver function profiles, with higher serum total bilirubin, alanine transaminase (ALT) and alkaline phosphatase (ALK-P) levels (175). Dey et al., showed that *Mycobacterium tuberculosis* can be an etiological factor for liver abscesses in HIV-infected patients (168). Infection by *Toxoplasma gondii* has also been reported to promote chronic liver disease in HIV-infected individuals (169). Hepatitis C virus infection is also known to act as an opportunistic disease in AIDS patients, directly causing progressive liver damage, which may also result in liver cirrhosis and hepatocellular carcinoma (176, 177).

Moreover, the medications associated with the drug treatment of opportunistic diseases are further contributing factors to persisting liver damage. The current first-line drug treatment for TB is a regimen of four drugs, i.e., isoniazid (INH), rifampicin (RIF), ethambutol (EMB), and pyrazinamide (PZA) (178). However, hepatotoxicity has been frequently observed as a serious adverse reaction following the use of these anti-TB drugs, especially with use of PZA, INH, and RIF, with a 2–28% incidence rate (179–183). Among PLWH, a higher incidence of hepatotoxicity has been seen, and Araújo-Mariz et al., have reported a 30.6% cumulative incidence rate of hepatotoxicity in PLWH following the use of recommended drugs for TB treatment (184). Sulfonamides, including trimethoprim/sulfamethoxazole (TMP/SMZ) and sulfadiazine, are other drugs which have been widely used in AIDS patients, and have been recommended as drugs of first choice for infections by *Pneumocystis jirovecii* and *Toxoplasma gondii* in HIV-infected patients (185). These drugs have also been frequently reported to induce hepatotoxicity (186–189).

Other contributing factors that may occur during the AIDS stage, such as paradoxical and unmasking immune reconstitution inflammatory syndrome (IRIS) and drug-drug interactions, may also result in liver disease or toxicity (190, 191). However, further studies of the baseline liver status of patients (uninfected by HBV) during the AIDS stage and studies of liver enzyme profiles in these patients during the AIDS stage are warranted to further assess other potential influencing factors for HBV establishment in patients with AIDS.

## Conclusion

It is known that HIV infection induces an immunodeficiency syndrome, rendering the patient vulnerable to infections, including HBV infection. The present review is the first to critically discuss the specific mechanisms leading to HBV establishment in a patient who is already HIV-positive. We report that the acute phase is responsible for a sudden immune system defense subversion, a CD4+ T-cell depletion, and a high viral RNA load, all contributing to increasing the vulnerability of the liver, which subsequently inexorably develops a permissiveness to HBV. During the chronic phase of HIV infection, gut-associated dysbiosis and immune cell exhaustion, compounded by the hepatotoxic phenomena encountered during the acute phase, are two major consequences of HIV infection which are likely to enhance the probability of subsequent HBV invasion of the liver. The other possible facilitatory causes for HBV invasion of the liver in HIV-infected patients that we have discussed herein are the use of modern ART, and HIV-associated B-cell depletion. Finally, the AIDS phase of HIV infection is often defined by particularly low CD4+ T-cell counts, OIs (and OI-related drug treatments), and extraordinarily high viral RNA loads, all of which, as we have described herein, conspire to inflict further sustained injury to the liver, which also favors HBV establishment.

## Author Contributions

SZ and JO wrote the first draft of the manuscript. YLC, YJ, and HW provided critical revision of the manuscript. YKC conceived and designed the study. All authors read and approved the manuscript and its submission for publication.

## Funding

This work was supported by the Chongqing Talent Cultivation Program (cstc2021ycjh-bgzxm0275), the Joint Medical Research Project (2020GDRC010) of Chongqing Science & Technology Bureau and Chongqing Health Commission, the Chinese Federation of Public Health foundation (GWLM202024), and the Chongqing Science & Technology Bureau project (cstc2020jscx-cylh0004).

## Conflict of Interest

The authors declare that the research was conducted in the absence of any commercial or financial relationships that could be construed as a potential conflict of interest.

## Publisher’s Note

All claims expressed in this article are solely those of the authors and do not necessarily represent those of their affiliated organizations, or those of the publisher, the editors and the reviewers. Any product that may be evaluated in this article, or claim that may be made by its manufacturer, is not guaranteed or endorsed by the publisher.
